# Mechanosensitivity of Cells and Its Role in the Regulation of Physiological Functions and the Implementation of Physiotherapeutic Effects (Review)

**DOI:** 10.17691/stm2020.12.4.10

**Published:** 2020-08-27

**Authors:** Yu.P. Potekhina, A.I. Filatova, E.S. Tregubova, D.E. Mokhov

**Affiliations:** Professor, Department of Normal Physiology named after N.Y. Belenkov; Privolzhsky Research Medical University, 10/1 Minin and Pozharsky Square, Nizhny Novgorod, 603005, Russia;; Student, Faculty of Pediatrics; Privolzhsky Research Medical University, 10/1 Minin and Pozharsky Square, Nizhny Novgorod, 603005, Russia;; Professor, Department of Osteopathy; North-Western State Medical University named after I.I. Mechnikov, 41 Kirochnaya St., Saint Petersburg, 191015, Russia; Associate Professor, Institute of Osteopathy; Saint Petersburg State University, 7/9 Universitetskaya naberezhnaya, Saint Petersburg, 199034, Russia; Head of the Department of Osteopathy; North-Western State Medical University named after I.I. Mechnikov, 41 Kirochnaya St., Saint Petersburg, 191015, Russia; Director of the Institute of Osteopathy Saint Petersburg State University, 7/9 Universitetskaya naberezhnaya, Saint Petersburg, 199034, Russia

**Keywords:** mechanosensitivity, mechanical transduction, mechanical stimuli, extracellular matrix, osteopathic manual therapy, mesenchymal stem cells.

## Abstract

Regulatory signals in the body are not limited to chemical and electrical ones. There is another type of important signals for cells: those are mechanical signals (coming from the environment or arising from within the body), which have been less known in the literature. The review summarizes new information on the mechanosensitivity of various cells of connective tissue and nervous system. Participation of mechanical stimuli in the regulation of growth, development, differentiation, and functioning of tissues is described. The data focus on bone remodeling, wound healing, neurite growth, and the formation of neural networks. Mechanotransduction, cellular organelles, and mechanosensitive molecules involved in these processes are discussed as well as the role of the extracellular matrix. The importance of mechanical characteristics of cells in the pathogenesis of diseases is highlighted. Finally, the possible role of mechanosensitivity in mediating the physiotherapeutic effects is addressed.

## Introduction

The mechanisms of biological regulation are usually discussed in terms of neuro-humoral pathways mediated by action potentials and physiologically active substances. However, there is another type of important signals for body cells, namely mechanical signals (coming from the environment or arising from within the body). Different types of motion (contraction of skeletal, myocardial, or smooth muscles, maintaining the posture and the like) create pressure and tension in various anatomical structures. The mechanical forces acting in the body are classified as tensile stress, compressive stress, vibration, hydrostatic pressure, and shear stress due to flow of fluids [1].

Connective tissue membranes (fasciae) form a single tensegrity system that encompasses structures of the human body. Starting from the connective tissue septa in the subcutaneous fat, the fasciae cover groups of muscle, individual muscles, and muscle fibers; together they form envelopes that cover internal organs, nerves, the spinal cord, and the brain. All these structures are interconnected, forming the fibrous skeleton of the body. Thus, using fasciae, all internal organs are connected with each other and with skeletal muscles [2]. Due to the continuity of the connective tissue skeleton, mechanical signals are transmitted along the fasciae to organs and tissues. Living organisms use the principle of tensegrity to mechanically stabilize their shape, as well as integrate and balance their structures at various architectural levels [3, 4].

The understanding that mechanical forces regulate tissue development and remodeling emerged more than a century. That time, Julius Wolff noted that bone trabeculae were adjusted to the main stress lines caused by daily physical activity; he then suggested that bone tissue was able to adapt its architecture to the mechanical environment (Das Gesetz der Transformation der Knochen, 1892). In the last quarter of the XX century, studies demonstrated that mechanical signals were specific for connective tissue [5, 6].

Mechanosensitivity is the ability of cells to perceive physical signals and mechanical forces generated in their microenvironment [7]. Research into biomechanical signals and mechanosensitivity lags behind most electrophysiological, molecular, and genetic studies. Progress in the biomechanical field often depends on the availability of appropriate experimental techniques. Not before recently, methods have been developed that provide for quantitative probing and controlling the mechanical parameters, such as the stiffness of tissues, cells, and subcellular structures, as well as the cellular tension forces. Most of these methods are based on direct contact; they are invasive and/or suitable only for *in vitro* studies [8]. For obvious reasons, studies of mechanical factors *in vivo* are less feasible.

Recent studies have shown that mechanical forces affect the growth and shape of almost all tissues in the human body. Deformations of tissues are transmitted via the extracellular matrix (ECM) to the cells. In the case of pluripotent cells, these processes control the subsequent differentiation [9].

The nature of the interaction between the cell and the ECM determines the degree of deformation, which can be weakened or strengthened [10], just like the nature of nuclear interactions with the cytoskeleton determines the degree of nuclear deformation in response to pressure or tension [11].

The authors’ task was to review the data on mechanosensitivity of cells in various organs and tissues, on mechanotransduction, and the role of mechanical stimuli in the regulation of physiological functions and the implementation of the effects of physiotherapy.

## The effect of mechanical signals on the connective tissue

It is known that deformation of connective tissues initiated by mechanical stress is able to induce the synthesis of structural biopolymers and thus modify the structure of the intercellular matrix [12]. Such a restructuring is necessary to maintain the adequacy between the viscoelastic properties of the tissue and the stress-induced tissue changes. When the connective tissue is stretched, the synthesis of collagen and elastin is activated; yet, the growth of collagen develops about three times faster, as it was found in the aortic wall [13]. Deformation of cells under mechanical stress induces the collagen synthesis. Under mechanical load, the transverse binding in collagen fibers weakens, and its solubility increases [14].

Serov and Shekhter with co-authors [5, 15] proposed the concept of “biomechanical control of morphogenesis”, according to which a fibroblast determines the micro-architectonics of its vicinity; likewise, a cell population determines the architecture of the entire tissue. The controlling mechanism in this process is the compatibility between the structure and the biomechanical function. Fibers that do not fit the lines of mechanical stress and do not, therefore, have any functional significance, are resorbed, while other fibers increase their volume until the “biomechanical compatibility” is reached. Thus, the process of tissue construction is facilitated by the crosstalk between cells and ECM.

It is known that the bones are continuously degraded and then restored in the process of remodeling; in this, osteoblasts form a new bone, and osteoclasts resorb the old bone. Although many factors, such as diet, hormone levels, and age, can tilt this balance towards the bone formation or resorption, mechanical stimuli are an essential factor in strengthening the bone structure; the dynamically changing mechanical environment is needed for the formation and maintenance of healthy bones [16]. Postnatal bone formation is controlled by osteogenic cells that respond to various mechanical stimuli [17]. The absence of mechanical stimuli (paralysis) or the absence of external mechanical load (during bed rest or in weightlessness) reduces the formation of the bearing bone and weakens the bone structure [18–20].

The outcomes of bone remodeling in response to mechanical stress depend on the recruitment of bone marrow mesenchymal stem cells (MSCs) into the osteogenic line. When physical activity is absent, MSCs tend to enter the adipogenic line [21] — the phenotype that prevails in paraplegic, inactive, and elderly people [22]. In the bone marrow, MSCs are located in close proximity to bone surfaces and are continuously exposed to mechanical signals induced by physical activity. When mechanosensitivity of these progenitor cells is impaired (with aging), mechanical signals cannot control the fate of these cells anymore, which ultimately leads to osteoporosis [23].

mesenchymal stem cells are mature multipotent cells with a powerful potential for self-renewal and differentiation into multiple cell lines; MSCs are derived from various mesenchymal tissues such as bone marrow, adipose tissue, umbilical cord and dermis [24, 25].

To direct their differentiation and proliferation, the participation of ECM (architectonics, rigidity, etc.) and external mechanical stimuli are important [26]. Depending on the intensity of deformation, MSCs can differentiate into cells of various types. For example, with bone marrow MSCs deformation of 3 or 10%, osteogenesis or tendon formation dominates [27]. The oscillatory fluid flow induces shear stress and, as it turns out, promotes both osteogenic and myogenic differentiation [28]. The compression load promotes the chondrogenic differentiation of MSCs and increases the expression of chondrogenic markers, such as collagen II and aggrecan [29]. Another study showed that these external mechanical signals could induce osteogenic differentiation of MSCs, increase bone matrix formation and calcium deposition [30]. Vibration promotes osteogenesis and increases the expression of osteogenic markers (osteopontin and osteocalcin) [31].

Wound healing is a complex multi-stage process, which involves cells of different types and requires strict regulation of biochemical and biomechanical signals [32]. Fibroblasts remodel the ECM within the wound, in order to secure the mechanical stability and provide the “substrate” for other cells and growth factors. In the early stages of wound healing, dermal fibroblasts infiltrate the lesion and secrete ECM proteins such as collagen and elastin. There is a bidirectional mechanical connection between the ECM and fibroblasts, which is mediated by integrins. Those are associated with intracellular mechanosensitive adapters and signaling proteins (see below) involved in remodeling of the ECM in response to mechanical stress. These bidirectional dynamic mechanical connections between the cell and the environment have are important for tissue structure and function; their disorders can contribute to the formation of keloids [33]. It is notable that areas of the skin that undergo periodic stretching and compression have an increasing trend to the development of keloid scars [34].

Thus, connective tissue cells are sensitive to various mechanical stimuli, which participate in the differentiation, reproduction, and functioning of these cells. For practical implications, by applying a calibrated tension, or, conversely, by removing it, one can change the architectonics of connective tissue and trigger its remodeling.

## The impact of mechanical stimuli on the development of the nervous system

Until now, the development of the nervous system has been considered, to a large extent, in the context of biochemistry, molecular biology, and genetics. It is commonly accepted that most neurons respond only to chemical signals. However, there is growing evidence that the nervous system is able to assimilate the mechanical information essential for the differentiation of neural precursors, for neuron migration, for the growth of axons and dendrites, and the formation of cortical gyri [8]. Studies *in vitro* have shown that many types of neurons and glial cells are able to respond to mechanical signals [35].

Neurons have long processes that are subjected to mechanical tension [36, 37]. On a larger scale, the whole neural tissue in developing organisms is subjected to tension [38]. In adults, the neural tissue is mechanically heterogeneous: there is a difference between the mechanical properties of the white and gray matter in the brain [35, 38, 39]. In addition, the stiffness of adult brain tissue increases with age [40]. During their development, the nervous tissues modify their mechanical properties so that cells encounter different mechanical signals depending on their location and stage of development. It can be assumed that at a certain stage of development, stiffness of the cortical tissue can exceed some critical threshold, thereby triggering the transition from neurogenesis to gliogenesis [8]. The increased expression of glial fibrillary acidic proteins in astrocytes increases the stiffness of the nervous tissue, which inhibits neurogenesis [41]. In contrast, the differentiation of Schwann cells and oligodendrocyte progenitor cells, which are glial cells, increases with increasing stiffness [42, 43].

The brain cortex folding in mammals facilitates the distribution of mechanical stresses in the gyri [44]. Neuroblasts begin to differentiate in the upper parts of gyri earlier than in the lower parts; these cells enlarge earlier, and their dendrites are much more developed, indicating that mechanical stress may be involved in the development of progenitor cells. Studies confirming this hypothesis demonstrate that mechanical stress leads to the differentiation of nerve stem cells towards mature neuronal cells *in vitro* [45].

**Figure 1:**
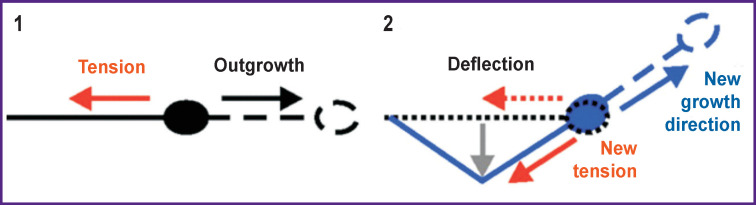
The distribution of forces during the growth of a neurite: *1* — initially, the growth cone moves (*black arrow*) in the direction opposite to the tension acting along the neurite (*red arrow*); *2* — when the neurite deflects (*gray arrow*), the force is redistributed and the neurite growth changes its direction so to resist the new tension [8]

Many types of neuronal cells adapt their morphology, specifically, the number, length and branching structure of their neurites, to the stiffness of their substrate *in vitro*, including ganglion cells of the mammalian spinal cord, neurons of the spinal cord and hippocampus, but not necessarily neurons of the cerebral cortex [46, 47]. The growth of neurites is a mechanical process, and as such it may well be due to the interaction between neurites and the mechanical environment *in vivo*. From the beginning of neurite growth to the establishment of synaptic connections with the target cell and the formation of stable neural networks, they are constantly subjected to mechanical stress [36, 48]. Stresses above or below a certain threshold stimulate the growth or retraction of neurites, respectively [37, 49] (Figure 1).

Pfister et al. [50] showed that mechanical tension induced extreme stretch growth of integrated axons with the amazing speed and length (8 mm/day). This result implies that axonal elongation is mainly limited by a lack of tension. Accordingly, when neurons are cultivated on a flexible substrate, the length of neurites increases significantly with increasing stretching of the substrate, and the neurites get aligned along the direction of the stretching [45].

Similarly, stress can influence the final morphology of neural networks. As soon as the neurite connects with its target, stress helps stabilize it; at the same time, it causes retraction or elimination of collateral neurites [51]. Thus, stress can serve as a signal for axonal and dendritic survival, and a decrease in stress can therefore contribute to growth cessation [52]. Once the neural network is interconnected, an increase in mechanical stress leads to shortening of the involved neurite, which contributes to the compactness of neural networks [53].

There is an assumption that stress promotes the formation of a synapse [49]. Experiments *in vivo* show that stress directed along the axon can be actively controlled by neurons and even participate in the functioning of synapses. For example, when *Drosophila* axons are stressed, neurotransmitter vesicles accumulate in presynaptic terminals of the neuromuscular junction [36]; in these conditions, stress modulates the local and global dynamics of the vesicles [54]. Consequently, mechanical stress in neuronal axons and along them can contribute not only to the formation of a neural network but, eventually, to the regulation of the neuron function.

The hypothesis of “differential expansion of the cerebral cortex” suggests a central role of mechanical forces arising in the process of cortical development. In this hypothesis, it is assumed that the tangential expansion of the cortical regions, which is caused by local cell proliferation and changes in cell size and shape, is the driving force for the formation of gyri and sulci [55]. According to another model, the cortex folding is caused not by the gray matter, but by stress developing in the white matter (the stress originates from the cortical-cortical and cortical-subcortical connections) [56]. Both mechanisms are not mutually exclusive and are likely to contribute jointly to the brain formation.

Thus, many events in the neuron development are, apparently, controlled by mechanical stimuli. Cell sensitivity to mechanical stimuli may be used as an additional level of control over the development and as a fundamental way of interacting with a changing environment. Consequently, the mechanical stress in the nervous system, especially in the process of its growth and development, may cause health disorders, and also act as a therapeutic factor.

## Mechanical transduction

Mechanical signals can travel from macro-structures to cells and subcellular organelles via the tensegrity system consisting of the ECM, cytoskeleton, and nuclear matrix, up to DNA [3].

Mechanical stimuli are transmitted to cells through the ECM — a structured macromolecular network that creates a scaffold for cell support and interaction [57, 58]. The matrix is made of fibrous proteins (collagen, elastin, laminin, fibronectin), glycosaminoglycans (e.g., hyaluronic acid), proteoglycans (chondroitin sulfate, heparan sulfate, keratan sulfate), and soluble components (cytokines, growth factors, and various proteases). All ECM components act as mediators that transmit mechanical signals to cells [57, 59, 60]. These signals may cause membrane deformation, which (if it is strong enough) can cause conformational changes in transmembrane proteins and even reach the cytoskeleton and nucleus [8].

**Figure 2:**
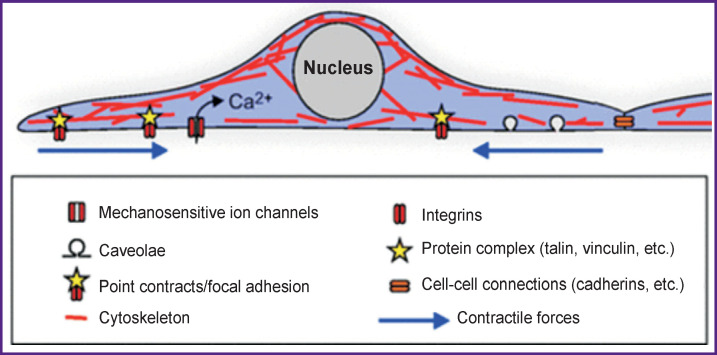
Possible tense-sensors in the cell [8]

Mechanotransduction is defined as the conversion of mechanical stimuli into an intracellular biochemical response. The molecular basis of cellular mechanotransduction is still poorly understood. The list of candidates for the role of cell tense-sensors includes the stretch-activated ion channels, caveolas, phosphorylation sites, cell adhesion sites (including cell adhesion molecules such as integrins and cadherins), proteins that bind these molecules to the cytoskeleton (vinculin and talin), signaling proteins (focal adhesion kinase), adapter proteins (p130Cas), cytoskeleton and the nucleus itself (Figure 2). Other possible key elements of mechanotransduction are direct physical effects and stress-dependent exocytosis and endocytosis [61–64].

Mechanical stretching of cell membranes changes the transport activity of mechanosensitive ion channels as a result of conformational changes or tension in the lipid bilayer [65] and the gate domains of the channel itself [66, 67]. As shown, most channels respond to cellular stretching, but not compression. Deformations may occur in calcium ion channels as well; there they induce changes in calcium permeability. Those, in turn, may interact with the signaling pathways that involve calcium as a second messenger [68]. For example, mechanosensitive calcium channels are thought to play a key role in chondrogenic differentiation of MSCs [69].

Integrin proteins (penetrating through the cytolemma) bind the cytoskeleton with the help of focal adhesive complexes and function as a direct connection between the ECM and the intracellular environment. Focal adhesive complexes — sensory elements that connect the cell plasma membrane with the extracellular matrix — play a decisive role in the perception of mechanical signals generated in the external milieu [70]. Nanotopography of the ECM can control cell behavior by changing the cell interaction with integrins and/or focal adhesive complexes.

Mechanical stimuli can be transmitted from the plasma membrane through the cytoskeleton directly to the nuclear membrane and its associated proteins. This process does not require biochemical signaling and can occur in a much shorter time scales (about 1 ms vs 5–10 s) [11]. In order to sense extracellular mechanical signals, the nucleus must be physically connected to membrane-bound focal adhesive complexes. In a study of Maniotis et al. [71], mechanical connection between the plasma membrane and the nucleus was demonstrated for the first time.

In all cells, the cytoskeleton acts as a dynamic machine that accumulates the external forces applied to the cell from the microenvironment and responds by generating tension/compression forces that are transmitted to other molecular components inside or outside the cells, including those that strengthen the cytoskeleton by creating new stress fibers [72]. This model is based on the concept of tensegrity, which helps living cells organize their cytoskeleton as a stiff wire that instantly responds to external mechanical stresses that stabilize its shape [73]. Integrin-bound microtubules and microfilaments get deformed, which leads to a reorientation of cytoskeletal filaments and redistribution of the nucleoli. These data suggest that an external mechanical force can not only deform the nucleus but also induce a reorganization of its genomic content, potentially regulating the gene expression. This distribution of force is mediated by both intermediate filaments and F-actin, which is the main player in the transmission of mechanical stimuli to the nucleus [1]. As a rule, tension is generated inside the contractile microfilaments of actomyosin; microtubules resist the compression forces [74]. Such mechanically controlled changes in the cellular structure not only provide an effective transmission of forces to the nucleus but also dynamically adjust the nucleoskeleton architecture and associated gene expression, thereby physically affecting the biological response [11].

It is known that during the differentiation of stem cells, the nuclear architecture consisting of chromatin and the nucleoskeleton, undergoes changes that vary in various somatic cells. Mechanical stimuli are important effectors of differentiation and cause stimulus-specific changes in nuclear architecture. This occurs during mechanotransduction, when extracellular mechanical forces activate the signaling cascades originated in the cytoplasm and nucleus [75].

Mechanical changes of the plasma membrane lead to subsequent nucleocytoplasmic movement of various transcriptional regulators. Signaling via Wnt/β-catenin is one such pathway [76]. This path involves translocation of stabilized β-catenin to the nucleus, where it binds to transcription factors and regulates the transcription of target genes; these genes, in turn, regulate differentiation and proliferation [77]. Signaling via Wnt/β-catenin was recognized as crucial for the generation of MSC and also for developing the skeleton, as well as for healing fractures [78].

Recent studies have shown that several microRNAs (evolutionarily preserved short non-coding RNAs) are sensitive to various mechanical stimuli and play a vital role in various physiological and pathological processes, including cell differentiation, proliferation, apoptosis, and cancer development. The accumulated data indicate that almost 40% of reports on exercise-sensitive microRNAs relate to skeletal muscle, and to a lesser extent, to bones *in vivo* [79]. MicroRNAs are sensitive to various mechanical stimuli when regulating the differentiation of osteogenic cells and the formation of bone tissue [80–82]. However, the functional role and mechanisms of mechanosensitivity of miRNAs are not fully understood. It has been established that mechanosensitive miRNAs participate in osteogenic differentiation by changing their expression under mechanical stimuli. Mechanosensitive microRNAs that serve as inhibitors of osteogenic differentiation have been described [79, 81]. Interestingly, the same microRNA can play opposite roles in the differentiation of various osteogenic cells subjected to various mechanical stimuli [83–85]. In aging, bone formation decreases. In [86], the miR-188 molecule was identified as a key regulator of age-related switching of the MSC differentiation from osteoblasts to adipocytes.

Recently, the nuclear membrane has also been recognized as a mechanosensory element regulating both biochemical and physical linkage between the nucleus and the cytoskeleton, as well as between the cell membrane and the ECM. A number of studies described the mechanisms by which the nuclear membrane and the associated proteins directly responded to extracellular mechanical perturbations [87–89].

In the nuclear membrane, there is a specialized structure known as the linker-complex of the nucleoskeleton and cytoskeleton (LINC), which provides a functional link between the supporting structures of the cytoplasmic and nuclear compartments [90]. The LINC complexes consist of domain-containing proteins Sad1/UNC-84 (SUN) located on the inner nuclear membrane and domain-containing proteins Klarischt/ANC-1/sine homology (KASH) located on the outer nuclear membrane [91]. LINC complexes allow the nucleus to sense signals from the extracellular mechanical environment, thus connecting the nucleus with actin and microtubules, and, consequently, with the ECM [92]. Suppression of the LINC complex and its associated nucleoskeleton (e.g., in aging) or a decrease in the mechanical stimuli, reduces the adaptive capacity of the cell and can contribute to the development of diseases such as osteopenia, sarcopenia, progeria, and obesity [9].

In the nuclear membrane, there is another structure with mechanosensitive properties: the nuclear pore complex (NPC), which mediates the passive and facilitated transport of substances between the nucleus and cytoplasm [93]. The current understanding of mechanosensitivity of the nuclear envelope and the role of NPC is yet to be improved [94]. At present, there are two theories of the mechanical opening of the pores. The first theory assumes that intracellular forces cause the nuclear envelope to stretch, thus increasing the pore size [95]. The second theory, which remains to be proved, is that cellular internal forces act on the nuclear part of the nuclear pore and on the basket, which is formed by eight nucleoplasmic threads, leading to rotational symmetry [96]. An external force emanating from the cytoskeleton and acting on the basket can expand the net and thus facilitate the passage of transcription factors accumulated in the basket. It is well known that in a cell subjected to mechanical stimulation, the flow of transcription factors into the nucleus increases [94].

In addition to direct mechanical stresses, the nucleus also responds to mechanochemical stimulation through the osmotic mechanism. Hypotonic medium induces the chromatin expansion and nucleus swelling, while hyperosmotic medium induces rapid condensation of chromatin [97], which increases the rigidity of the nucleus [98]. Tissue damage brings about osmotic swelling of cells and nuclei at the edge of the wound. Induced by this swelling, the nuclear membrane stretches and activates the inflammatory signaling cascade via enzyme-lipid interactions. These results show that the nucleus can directly respond to mechanical stimuli; notably, changes in both the regulation of genes and the mechanical properties of the nucleus itself are independent of biochemical reactions in the cytoplasm [99].

Cell mechanosensitivity depends on the mechanical properties of the cell and its components. Thorpe and Lee [75] suggest that as a cell responds to a mechanical stimulus or changes its function (e.g., during differentiation or disease), the mechanical properties of both the nucleus and the cytoskeleton also change to provide additional mechanosensitivity. It has been demonstrated that multiple mechanical impacts on the ECM, sensitize the cell to subsequent mechanical stresses [98, 100]. Several consecutive episodes of mechanical deformation cause a state of chromatin condensation, which persists for at least 5 days in the absence of further deformation. This condition of “enhanced chromatin condensation” may increase the nuclear stiffness and also the cytoskeleton stiffness, which may activate specific mechanosensory mechanisms in the cell. Mechanical impacts reversibly increase the number of focal adhesions and associated cytoskeletal structures aiming to adapt to subsequent mechanical perturbations [101].

Thus, elements of the cell membrane, cytoskeleton, and nucleus participate in mechanotransduction. The origin of the mechanotransduction in the cell can influence the biochemical response to mechanical stimuli. Cellular mechanosensitivity depends on its own mechanical properties, and they, in turn, can change under the influence of mechanical stimuli. Similar to the chemical signaling pathways, more than one mechanism is involved in cellular mechanotransduction. In addition, individual mechanical and chemical signals can activate the related descending signal paths and thus interact with each other [68]. However, the problem of mechanical perception by various cells requires further study.

## The use of mechanical effects for treatment

**Figure 3:**
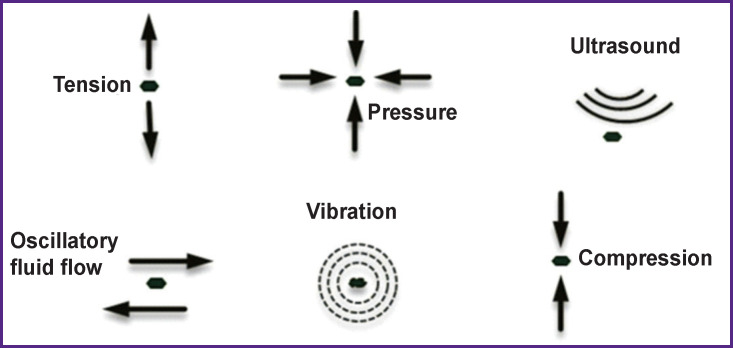
Various mechanical effects on tissues and organs [1]

The above discussion suggests that mechanical effects on the body (Figure 3) can have a therapeutic significance in a wide range of diseases.

At the end of the last century, it was shown that regular dosed movements helped collagen fibers to get arranged in the most optimal way so to create an adequate type of connective tissue and minimize the scar growth [102]. Physical or therapeutic load can reduce the number of intramolecular cross-links between alpha chains of collagen and intermolecular cross-links between collagen fibrils, filaments, and fibers, thus stimulating collagen metabolism [14].

It is well known that physical exercise significantly improves the properties of the trabecular bone, its mineral density, and mechanical strength in the natural process of bone growth. Periodical (preferably daily) physical activities, such as walking, running, cycling, or swimming are especially effective for the prevention of osteoporosis [103, 104]. Notably, cyclic deformations of the skeleton (i.e. intermittent compression and stretching) inhibit adipogenesis and stimulate osteo- and chondrogenesis [105]. Cyclic exercise has been shown to reduce the risk of degenerative osteoarthrosis/osteoarthritis compared with a sedentary lifestyle [106, 107]. Properly selected exercises delay the need for joint replacement surgery, reduce the need for prosthetics by 44%, and reduce the symptoms of osteoarthrosis/osteoarthritis at any age [108–110]. Therefore, regular physical activity positively influences the state of bone tissue and the joint movement; this well-known phenomenon can be used for the prevention and treatment of degenerative diseases of the musculoskeletal system.

The use of high-frequency, low-intensity vibrations (oscillations) imitates the effects of physical exercises and improves the function of the musculoskeletal system [111]. Oscillations stimulate osteogenesis in MSCs and slow down their conversion to adipocytes in the bone marrow [112]. Low-intensity pulsed ultrasound activates the chondrogenic differentiation of rat MSCs, promoting the formation of ECM and increasing the expression of chondrogenic markers [113]. The use of low-intensity vibrations can promote wound healing in diabetic mice [114].

The LINC complex is crucial for the perception of high-frequency low-intensity oscillations that regulate MSCs differentiation [9]. Low intensity vibration activates the focal adhesion kinase and subsequent remodeling of the cytoskeleton in MSCs, increases remodeling of F-actin in the perinuclear region, potentially modifying the mechanical connection throughout the cell. Mechanical treatments, such as low intensity vibration, first stimulate the formation of a strong connection between the cytoplasmic and nuclear cytoskeletons thus making the cell more sensitive to mechanical or biochemical signals in general. Not surprisingly, the use of low intensity vibration enhances the response to other mechanical and biochemical factors, including repeated exposure to low intensity vibration, which is more efficient than one-time exposure in suppressing adipogenesis in MSCs [31, 114, 115]. Extrapolating the above data to the organism level, it becomes obvious that short but repetitive workouts are more effective in achieving high results than a single long workout [116].

In experimental studies on cell mechanosensitivity and mechanotransduction, relatively weak mechanical impacts are used (3–4 g/cm^2^); those cause cell deformations by 10–12% of its initial size [117–119].

Manual treatment methods, including osteopathic manual therapy (OMT), can also be attributed to mechanical effects on the body. Osteopathy is a field of clinical medicine based on a systematic approach and using manual methods at all stages (prevention, diagnosis, treatment, and rehabilitation) of providing medical care to patients with somatic dysfunctions, restoring the body’s ability to self-repair [120]. OMT is a form of physical action on the body aiming to eliminate somatic dysfunctions and improve the health and functions of the body. Somatic dysfunctions are a potentially reversible structural and functional disorder of tissues and organs, manifested by palpation-determined limitations of movements and mobility [121]. The typical diagnostic indicators of somatic dysfunctions determined by palpation are biomechanical disorders: abnormalities of tissue texture (viscosity, elasticity, and rigidity), asymmetry, limitations of movement, and mobility [122]. Several models of somatic dysfunctions are proposed; in each of them, the tense-integrated fascia system acts as the main interface between the body systems, thereby providing a structural and functional basis for its homeostatic potential and the realization of innate healing abilities [123–125].

Doctors practicing osteopathy use a wide range of manual methods of mechanical action on various body parts, which can be classified as structural (effects on the musculoskeletal system), visceral and cranial. A common feature of osteopathic techniques is the soft, non-damaging, and painless impact on tissues aimed at restoring mobility [126–128]. The efficacy of OMT is explained by the involvement of mechanoreceptors [129], fasciae, joints, and muscles [130]. It has been suggested that manual touches can be transmitted (via the ECM) to connective tissues and stimulate their responses, including those delayed due to stress relief in the area of somatic dysfunctions. Fibroblasts sensitive to mechanical signals serve as a tool necessary for understanding of the therapeutic effect of OMT [130, 131].

*In vitro* modeling of controlled mechanical effects demonstrates how OMT can influence the behavior and proliferation of fibroblasts and participate in the inflammatory response (reduced interleukin secretion) [132, 133]. Currently, we know little about the function of fibroblasts in the presence of OMT. Further research is needed to better understand their behavior under the influence of OMT and choose the best osteopathic approach to manual treatment.

Regarding the cranial osteopathic techniques that are known to improve the CNS function, especially in young children [120] and trigger the nerve mechanotransduction, no relevant reports have been found in the available literature. The mechanical effects on the nervous system *in vivo* are extremely difficult to study. These are studies of the future.

## Conclusion

In order to survive, living organisms must feel, react and ultimately adapt to their physical environment at the cellular, tissue, organ, and organism levels. Adaptation is initiated at the cell level, where mechanosensory complexes develop, allowing for translating mechanical signals into biologically significant reactions. In the recent decade, significant progress has been made in identifying the mechanisms, by which cells perceive and respond to both static and dynamic mechanical signals, initiating signaling events that lead to differential gene expression and changes in the cytoskeleton [134]. These results indicate the ability of cells to distinguish between different mechanical signals from various sources.

All cells have developed structures that allow them to recognize mechanical signals and respond to them [135, 136]. At present, we know that mechanical stimuli control biological functions, including differentiation, reproduction, functioning, and metabolism [137, 138]. This adjustment is naturally dependent on the forces acting on the cells (without which there would be no motion) and by the resistance of cells and cell groups to these forces (the resistance depends on the cell viscoelasticity). These fundamental parameters have so far been largely ignored; today, it is clear that they are important for the understanding of the development processes as a whole [8] and explaining the effects of mechanical treatments.

Manual treatment methods (massage, manual therapy, OMT, etc.) exert the mechanical effects on the patient’s body. The weaker these effects (OMT), the more regulatory they are, causing a wide range of therapeutic effects. Studies on mechanosensitive molecules and the cytoskeleton can help discover novel therapeutic strategies for treating diseases with mechano-biological elements [139].
